# A biomimetic peptide has no effect on the isotopic fractionation during in vitro silica precipitation

**DOI:** 10.1038/s41598-021-88881-6

**Published:** 2021-05-06

**Authors:** Lucie Cassarino, Paul Curnow, Katharine R. Hendry

**Affiliations:** 1 University of Bristol, School of Earth Sciences, Wills Memorial Building, Queen’s Road, Brsitol, BS8 1RJ UK; 2 University of Bristol, School of Biochemistry, Medical Sciences Building, University Walk, Bristol, BS8 1TD UK

**Keywords:** Peptides, Marine chemistry

## Abstract

The stable isotopic composition of diatom silica is used as a proxy for nutrient utilisation in natural waters. This approach provides essential insight into the current and historic links between biological production, carbon cycling and climate. However, estimates of isotopic fractionation during diatom silica production from both laboratory and field studies are variable, and the biochemical pathways responsible remain unknown. Here, we investigate silicon isotopic fractionation through a series of chemical precipitation experiments that are analogous to the first stages of intracellular silica formation within the diatom silicon deposition vesicle. The novelty of our experiment is the inclusion of the R5 peptide, which is closely related to a natural biomolecule known to play a role in diatom silicification. Our results suggest that the presence of R5 induces a systematic but non-significant difference in fractionation behaviour. It thus appears that silicon isotopic fractionation in vitro is largely driven by an early kinetic fractionation during rapid precipitation that correlates with the initial amount of dissolved silica in the system. Our findings raise the question of how environmental changes might impact silicon isotopic fractionation in diatoms, and whether frustule archives record information in addition to silica consumption in surface water.

## Introduction

Silicon (Si) is an important nutrient in the biology of organisms such as diatoms, sponges, radiolarians and silicoflagellates. These organisms are capable of a process known as biosilicification, in which solid silica is precipitated in a controlled manner from dissolved silicon (dSi) acquired from the environment. Marine diatoms are generally heavily silicified, and are of particular interest since they are responsible for about 40% of primary production and a significant proportion of carbon export to the seafloor^1^. Many diatom species require Si for cell growth, meaning that there is a direct link between the global silica cycle, carbon uptake and climate change^2–4^.

**Figure 1:**
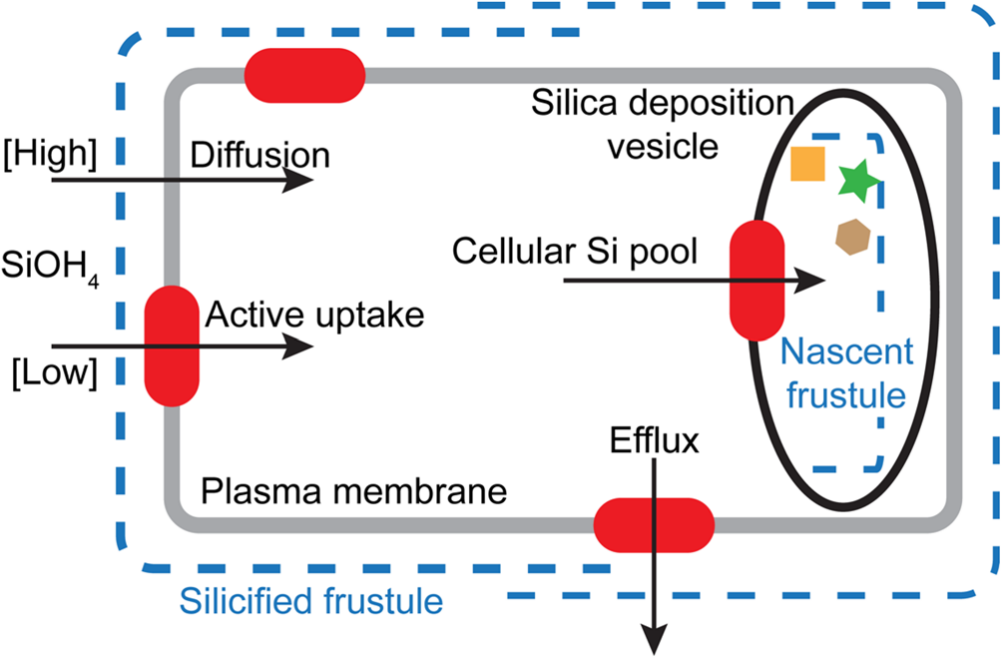
Schematic cartoon of silica formation in the diatom. Soluble silicic acid enters the diatom cell through active or passive transport and is moved to the silica deposition vesicle (SDV) via intracellular Si pools. The SDV is the site of frustule synthesis and the nascent frustule forms in the presence of various biomolecules, shown here as geometric shapes. See text for details.

Diatoms are encased in an outer cell wall, or frustule, made of hydrated amorphous silica ($\hbox {SiO}_{2}\cdot$*n*$\hbox {H}_{2}\hbox {O}$). This is often referred to as opal or biogenic silica (BSi)^3,5^. The process of diatom silicification is still not well-understood, but it appears to occur inside the cell through a series of tightly-controlled, coordinated and interdependent steps. Figure 1 briefly summarises the pathway of silicification in the diatom. The porous silicified outer cell wall (frustule) is formed of two overlapping halves (thecae). Soluble silica in the environment as monomeric silicic acid, Si(OH)$_{4}$, moves through the porous frustule and across the underlying plasma membrane via passive or active transport. The cellular Si pool, which can approach high millimolar concentrations (150 mM^6^), is likely balanced by efflux processes^7^. This accumulated silica is then transferred to the intracellular silicon deposition vesicle (SDV) which is the site of frustule formation. The SDV is likely to be a golgi-derived vesicle with an acidic lumen^8^. Biomineralisation occurs within the SDV through polymerisation^9^. The SDV probably contains genetically-encoded, species-specific biomolecules that are thought to support and direct silica biosynthesis, and which are found intimately associated with the mature frustule. These biomolecules are represented as geometric shapes in Fig. 1 and include long-chain polyamines^10^, as well as the different peptides known as silaffins, silacidins and frustulins^11–13^. Other factors, such as the SDV membrane and the cytoskeleton, are also thought to be critical in forming the structure of the frustule^9^ but are not shown or discussed further here. The final stage of silicification involves the export of the nascent frustule to the cell exterior and subsequent maturation. While these general principles of biomineralisation are known to a certain degree, many of the specific processes underlying them remain poorly understood.

Previous studies have attempted to understand the role of SDV-localised biomolecules by studying their ability to influence the formation of silica in vitro^14–16^. An attractive model system in this regard is the peptide known as ‘R5’, which has the sequence NH2-SSKKSGSYSGSKGSKRRIL-CO2H and was derived from the sequence of a silaffin peptide from *Cylindrotheca fusiformis*^17^. The native silaffin on which R5 is based is rather complex, featuring extensive and unusual posttranslational modifications, and must be extracted from diatom silica^17,18^. In contrast, R5 can be obtained easily by facile peptide synthesis. The major drawback of using R5 is that while the native silaffins are highly active at low pH, consistent with the likely environment of the SDV lumen, the synthetic R5 peptide is only active at pH >7^12,19^. Nonetheless R5 has found favour as a tractable surrogate for understanding how short, charged peptides might influence silicification^20–24^. The precise mechanism of interaction between R5 and Si is unknown and is still actively under debate. However, previous studies have suggested that as the solid silica phase starts to precipitate and accumulate negative charge, this is compensated by the adsorption of the positively-charged peptide to the silica interface. This electrostatic effect seems likely to be supported by other interfacial interactions such as hydrogen bonding. Cumulatively, these interactions facilitate precipitation and lead to R5 becoming trapped within the solid^25,26^.

One interesting aspect of biosilica formation in the diatoms is the bias towards lighter isotopes of silicon within the mineral^27–31^ This preferential incorporation of the light isotope ($^{28}$Si) leaves the heavy isotopes ($^{29}$Si and $^{30}$Si) in the surrounding seawater. This phenomenon is sufficiently robust that it can been used as a proxy for the biological consumption of dSi by key producers such as diatoms^29,31,32^. Measuring the Si isotopic composition ($\delta ^{30}\text {Si}$) of laboratory diatom cultures, samples from the modern ocean and sedimentary archives has thus provided a deeper understanding of the role of diatoms in the past and present Si cycle^33–36^. However, the biological mechanisms and pathways driving the fractionation of Si isotopes during biomineralisation are as yet unknown.

The true isotopic fractionation factor of diatom silica $\alpha$ is defined as:

1 $$\begin{aligned} \alpha _{A-B} = \frac{R_{A}}{R_{B}}, \end{aligned}$$

where R = $^{30}$Si / $^{28}$Si of component A (diatom) and B (dSi in seawater). This can also be expressed as the apparent fractionation factor, which is the difference between the Si isotopic composition of the diatoms and the seawater ($\Delta ^{30}\text {Si}_\text {p-s}$ = $\delta ^{30}\text {Si}_\text {diatom}$ - $\delta ^{30}\text {Si}_\text {dSi}$). Two models are commonly applied to determine the fractionation factor value from either laboratory or field data: the Rayleigh model (single input of silicic acid to a stratified system) and the open system model (continuous flux of silicic acid to a mixed system^27^). To date we have successfully used both Si isotopic fractionation models to study the role of organisms such as diatoms, sponges and choanoflagellates^31,37^ in the global Si cycle.

Several lines of evidence now point to both genetic and environmental control of isotope fractionation. Although early work reported consistent fractionation factors of $\epsilon$ = − 1.1 $\textperthousand$^27^, more recent studies have reported a range of fractionation factors across different diatom species^38^. The composition of other biosilicas, for example from sponges, is also markedly different from that of the diatoms, despite the mineral being chemically equivalent^37^. The same species of diatom can also display different degrees of fractionation depending upon the ambient conditions^38,39^. Finally, theoretical predictions of Si isotopic fractionation together with isotopic equilibrium experiments have shown extreme Si fractionation can occur during organosilicon complexation^40,41^. The interplay between environmental conditions and biochemical pathways that control fractionation is thus of keen interest for the basic understanding of diatom biology and for the robust interpretation of sedimentary geochemical archives of past ocean change.

A reasonable starting point is the assumption that fractionation might be occurring at the point of silica precipitation in the SDV, since this involves intimate engagement of the forming mineral with a complex assortment of biomacromolecules. The SDV has evaded detailed characterisation for decades, so we chose instead to study Si isotopic fractionation using the R5 model for silica precipitation in vitro. We investigated here whether the presence of R5 during Si precipitation was able to influence the Si isotopic composition of the resulting silica due to its role in the mediation of siloxane bonds^25^. We find that the presence of R5 does induce a systematic but non-significant difference in fractionation behaviour. Our results suggest instead that the isotopic composition of silica is largely driven by an early kinetic fractionation during rapid precipitation that evolves towards an equilibrium state. These findings imply that the SDV could be a site of Si isotopic fractionation within the diatom cell, and that this fractionation may not be substantially affected by co-precipitation with organic macromolecules.

## Results

### Equilibrium versus kinetic fractionation

Silica precipitation was induced by diluting sodium silicate from commercial stock solution into a well-defined saline buffer known as ND96 at room temperature (see “Methods”). We chose this particular buffer solution because it maintained a pH range in which R5 was active, included several biological salts, and, importantly, our initial tests confirmed that the buffer matrix did not interfere with the mass spectrometry analysis. The ND96 solutions were confirmed to have $\delta ^{30}\text {Si}_\text {dSi}$ very close to 0 $\textperthousand$. As expected, silica precipitation in this buffer was instantaneous at Si concentrations above approximately 2 mM, consistent with the known saturation point of silica of 1.93 mM at 25 $^{\circ }$C^42^. The precipitated silica was isolated at various timepoints by low-speed centrifugation, stopping the reaction and generating two fractions: a silica pellet, and a supernatant containing buffer salts and the remaining soluble Si. Measurement of soluble Si by molybdate assay was performed after each sub-sampling event and at the end of the experiments both fractions were sub-sampled for Si isotopic analysis.

**Figure 2:**
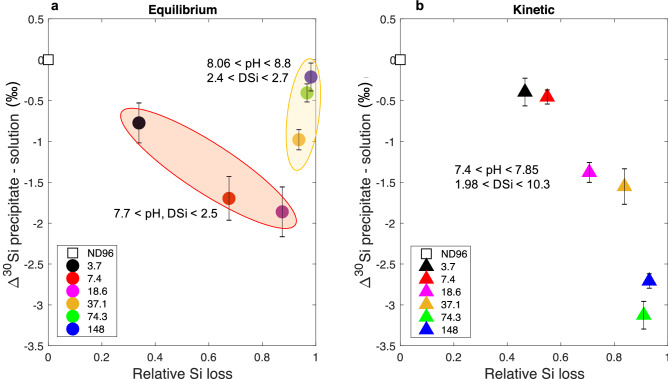
Si isotopic fractionation ($\Delta ^{30}\text {Si}_\text {p-s}$) as a function of the relative silicon loss from solution due to precipitation for a range of dissolved Si concentrations (mM). (**a**) Experiments after 8 days incubation. (**b**) Experiments after 1 h incubation. Each data point represents a different initial starting concentration of Si, coloured according to the key shown in each panel. Shaded areas in each panel group samples by comparable final conditions as shown. Error bars shows the 2 s.d. of the repeated measurements. The starting concentration 371 and 743 mM data are presented in Supplementary Table S1.

In a first set of experiments, the precipitation reaction was incubated for 8 days before the pellet and supernatant fractions were obtained and analysed. We assume that this is sufficient time for the reaction to have reached chemical equilibrium based on preliminary testing (Supplementary Fig. S1) and equilibrium isotopic fractionation (attained a constant rate of isotopic two way transfer between precipitate and solution) and so refer to these data as being at ‘equilibrium’. In a second set of experiments we separated the precipitate 1 h after mixing. We assume that these samples have not yet attained complete thermodynamic equilibrium and so refer to this as a ‘kinetic’ experiment. Figure 2 shows the Si isotopic fractionation between the precipitate and the solution of these equilibrium and kinetic experiments. This is presented as a function of the relative Si loss from the initial starting concentration (3.7–743 mM) due to precipitation.

In the equilibrium experiments, the silica precipitate generally had a negative $\delta ^{30}\text {Si}$ ($\delta ^{30}\text {Si}_\text {p}$) and the soluble Si had a positive $\delta ^{30}\text {Si}$ ($\delta ^{30}\text {Si}_\text {s}$) (Supplementary Table S4), indicating the preferential incorporation of lighter isotopes into the precipitate. This can be expressed as $\Delta ^{30}\text {Si}_\text {p-s}$  which is the difference between $\delta ^{30}\text {Si}_\text {p}$ and $\delta ^{30}\text {Si}_\text {s}$, and generally gives a negative number during silica precipitation^43–45^. The starting concentrations above 148 mM did not attain equilibrium likely due to high pH values (pH > 10). The two higher starting concentrations resulted in a $\Delta ^{30}\text {Si}_\text {p-s}$ equal to 0 $\textperthousand$ or positive, and so these were excluded and 148 mM was set as our upper concentration limit (Supplementary Table S1). As expected, in all other equilibrium experiments the concentration of dSi remaining in the supernatant after 8 days was just above the saturating concentration of 2 mM, and pH was consistently maintained in the range 7.7–8.8 (Fig. 2 and Supplementary Table S1). The initial pH values correspond to the pH of the kinetic experiment due to the instantaneous precipitation when silicate solution is added to the media (Fig. 2 and Supplementary Table S1).

**Figure 3:**
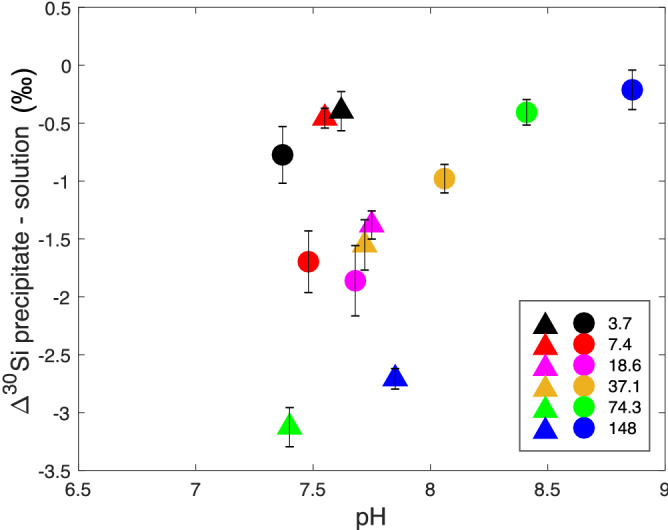
Si isotopic fractionation ($\Delta ^{30}\text {Si}_\text {p-s}$) as a function of pH during the initial kinetic (triangles) reaction and at equilibrium (circles) for the range of dissolved Si concentrations 3.7–148 mM. Data are also presented in Supplementary Table S1.

In contrast, the kinetic experiments showed a range of supernatant dSi concentrations (2–10.3 mM), indicating that in most cases we had successfully stopped precipitation before reaching a chemical equilibrium. The pH of the supernatant was between 7.4 and 7.9. The Si isotopic fractionation in these samples followed the same trend as for equilibrium samples, with preferential precipitation of the light Si isotopes. In comparison with the equilibrium experiment, here the $\Delta ^{30}\text {Si}_\text {p-s}$ shows a continuous trend towards more negative $\Delta ^{30}\text {Si}_\text {p-s}$ values with the relative amount of Si lost from the starting solution (Fig. 2b, Supplementary Table S1). At 3.7 mM the $\Delta ^{30}\text {Si}_\text {p-s}$ of the kinetic experiment is within the error range of the equilibrium $\Delta ^{30}\text {Si}_\text {p-s}$ at the same starting concentration. This observation is consistent with the similar relative Si loss between the two experiments for the 3.7 mM starting concentration. Previous studies have shown that pH can impact the stability and structure of silica precipitates^46^, and that at high pH (pH $\sim$ 9) the Si isotope exchange rate between precipitate and solution is higher, and so can affect the fractionation of Si isotopes by over 1 $\textperthousand$^47^. However the lack of correlation between pH and $\Delta ^{30}\text {Si}_\text {p-s}$ (Fig. 3) indicates that the range of pH across our experiments does not contribute significantly to the $\Delta ^{30}\text {Si}_\text {p-s}$ results.

Figure 3 shows the relationship between pH and $\Delta ^{30}\text {Si}_\text {p-s}$ for both equilibrium and kinetic experiments. At the range of pH in both experiments (7.37–8.86) the dominant silicon species is H$_4$SiO$_4$ with up to only 10% of silicon in the form of H$_3$SiO${_4}^{-}$, given the known speciation of aqueous Si species in equilibrium with amorphous silica as a function of pH^47^. The equilibrium experiment shows a negative correlation between pH and $\Delta ^{30}\text {Si}_\text {p-s}$ for pH < 7.7 and is followed by a positive relationship for pH > 7.7. In contrast the kinetic experiments have a narrower range of pH and do not show any obvious link between pH and $\Delta ^{30}\text {Si}_\text {p-s}$.

### Precipitation rate and the influence of biomolecules

**Figure 4:**
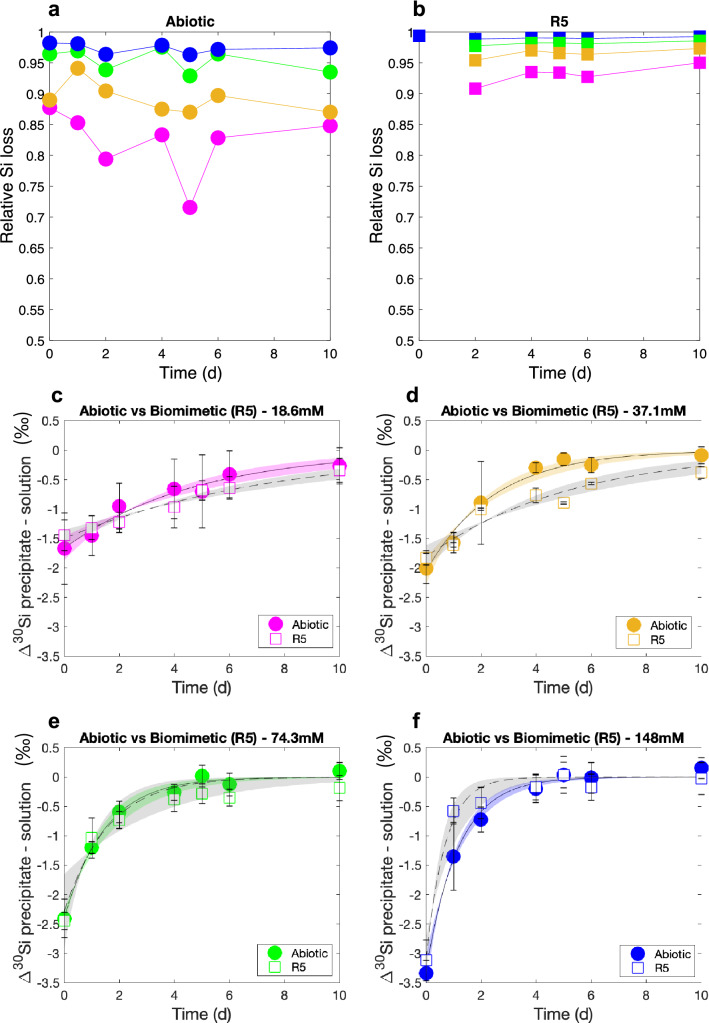
(**a**,**b**) Relative Si loss and (**c**–**f**) Si isotopic fractionation ($\Delta ^{30}\text {Si}_\text {p-s}$) as a function of time comparing the Abiotic (circles) and biomimetic (squares) experiment for initial dSi concentration of 18.6 mM (pink), 37.1 mM (orange), 74.3 mM (green) and 148 mM (blue). Solid and dashed lines show the best fit (f(x)=a exp(− bx)) with the 95% confidence bounds for the abiotic (coloured) and biomimetic (grey) experiments, respectively. The best fit equations are presented in Table 1. $\delta ^{30}\text {Si}$values are presented in Supplementary Tables S5 and S6.

We next investigated the impact of a mineralising macromolecule, R5, on Si isotopic fractionation. Figure 4a,b shows the variation in Si precipitation and $\Delta ^{30}\text {Si}_\text {p-s}$ for the abiotic (no R5) and biomimetic (R5) reactions at four concentration regimes over time. Data for dSi under abiotic conditions are from the colorimetric molybdate assay for soluble silica. dSi concentrations could not be determined using a colorimetric assay for the biomimetic experiment because the R5 peptide reacted with the molybdate reagent, and so were extrapolated from the Multi Collector Induced Coupled Plasma Mass-Spectrometer (MC-ICP-MS) voltages (see “Methods”). The potential interference of R5 on Si isotopic analysis was monitored by rigorous examination of mass bias, by tracking the mass-dependent behaviour of all stable silicon and magnesium isotopes^48^ (see “Methods”). Figure 4 also shows the best fit and the 95% confidence bounds considering data point error of the abiotic and biomimetic (R5) $\Delta ^{30}\text {Si}_\text {p-s}$ data series, with the best fits following the exponential function $f(x) = a \cdot \exp ^{\text {(-bx)}}$, which is in accordance with the temporal isotopic fractionation evolution of other light isotope systems during precipitation^49^. Table 1 presents the equations and error for the best fit from Fig. 4.

**Table 1:** Results of the curve fitting of the Si isotopic fractionation during precipitation ($\Delta ^{30}\text {Si}_\text {p-s}$) over time for the abiotic and the biomimetic (R5) experiment.

Initial dSi	Abiotic	Biomimetic (R5)
18.6 mM	$\Delta ^{30}\text {Si}_\text {p-s} = -1.67 \cdot \exp ^{{(-0.21 \; \text {day})}}$	$\Delta ^{30}\text {Si}_\text {p-s} = -2.31 \cdot \exp ^{\text {(-0.07 day)}}$
	$\hbox {r}^{2}$ = 0.96, RMSE = 0.05	$\hbox {r}^{2}$ = 0.97, RMSE = 0.03
37.1 mM	$\Delta ^{30}\text {Si}_\text {p-s} \ = -2.06 \cdot \exp ^{{(-0.41\; \text {day})}}$	$\Delta ^{30}\text {Si}_\text {p-s} \ = -1.81 \cdot \exp ^{\text {(-0.19 day)}}$
	$\hbox {r}^{2}$ = 0.98, RMSE = 0.03	$\hbox {r}^{2}$ = 0.97, RMSE = 0.02
74.3 mM	$\Delta ^{30}\text {Si}_\text {p-s} \ = -2.40 \cdot \exp ^{{(-0.69\; \text {day})}}$	$\Delta ^{30}\text {Si}_\text {p-s} \ = -2.29 \cdot \exp ^{\text {(-0.63 day)}}$
	$\hbox {r}^{2}$ = 0.99, RMSE = 0.03	$\hbox {r}^{2}$ = 0.90, RMSE = 0.07
148 mM	$\Delta ^{30}\text {Si}_\text {p-s} \ = -3.31 \cdot \exp ^{{(-0.86\; \text {day})}}$	$\Delta ^{30}\text {Si}_\text {p-s} \ = -3.11 \cdot \exp ^{\text {(-1.43 day)}}$
	$\hbox {r}^{2}$ = 0.99, RMSE = 0.05	$\hbox {r}^{2}$ = 0.98, RMSE = 0.09

These data show that the addition of R5 generally resulted in an increase of the relative Si loss compared to the abiotic experiment for the range of concentrations tested over the timescales of days (Fig. 4). However, this might simply be a function of the different ways that dSi was determined in these samples. Initially, day 0 (1 h) and day 1, $\Delta ^{30}\text {Si}_\text {p-s}$ was marginally less pronounced (less negative) in the samples containing R5. After the first day, fractionation was consistently slightly higher (more negative) in the samples containing R5, and this was most obvious at the earlier timepoints. This suggests that the presence of R5 could subtly increase isotopic fractionation during silica formation over the shortest timescales tested. The exception to this was at the highest dSi concentration, where fractionation in the presence of R5 was less than in the abiological sample. Despite these systematic observations, the experiments with and without R5 are not significantly different over the all length of the precipitation (within 95% confidence bounds). Overall, then, our experiments suggest that the presence or absence of R5 makes little difference to the initial fractionation or subsequent exchange behaviour of silica in vitro.

**Figure 5:**
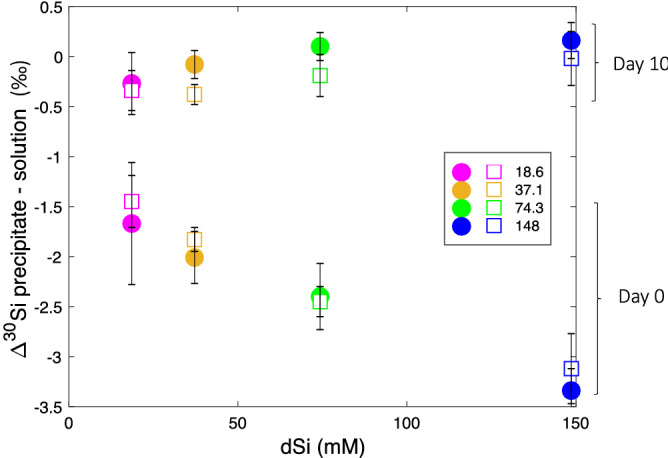
State (day 10) for the abiotic (circles) and biomimetic (squares) precipitation for the four different Si concentrations, 18.6 mM (pink), 37.1 mM (orange), 74.3 mM (green) and 148 mM (blue).

Figure 5 focuses on the difference between the early (kinetic, day = 0) and late (equilibrium, day = 10) phase of the precipitation presented in Fig. 4. During the kinetic phase $\Delta ^{30}\text {Si}_\text {p-s}$ and the Si concentration (from silica addition) are negatively correlated for both the abiotic and the biomimetic (R5) experiments. In contrast, at steady state, $\Delta ^{30}\text {Si}_\text {p-s}$ and Si are positively correlated. During both phases, there is a consistent but non-significant difference of 0.14 $\textperthousand$ between abiotic $\Delta ^{30}\text {Si}_\text {p-s}$ and R5 $\Delta ^{30}\text {Si}_\text {p-s}$.

## Discussion

The new experimental data presented here further support observations that simple chemical systems can reproduce the Si isotopic fractionation factors observed in diatom silica^29,31,38,50,51^. Our results confirm that lighter isotopes of Si are preferentially incorporated into silica during the process of precipitation, and we reproduce the change from initial isotopic kinetic fractionation toward equilibrium exchange that has previously been observed for Si and in other isotope systems^43,44,49^. Our results are also in accordance with Oelze et al., 2014 since the extent of the initial kinetic fractionation is inversely correlated to the relative Si loss (Fig. 2), i.e. the greater the proportion of the initial dSi that precipitates, the greater the observed fractionation will be. This means that fractionation is more pronounced for samples with higher initial starting concentrations of Si (Fig. 4, Table 1). Because of differences in experimental settings between the equilibrium/kinetic (Fig. 1) and the abiotic/biomimetic experiments, the following discussion focuses on the $\Delta ^{30}\text {Si}_\text {p-s}$ trends with Si concentration and relative Si loss.

The presence of various macromolecules, such as charged peptides like R5, could potentially influence Si isotope fractionation on biologically-relevant timescales. This is reinforced by the observation that the addition of R5 can increase the rate and yield of silica precipitation^19^. He et al. and Stamm et al. showed that the presence of organic compounds can induce large Si isotopic fractionation ($< -$10 $\textperthousand$) via the formation of hyper-coordinated organosilicon complexes. However, in the model system employed here we found no significant change in fractionation behaviour upon the addition of R5. Furthermore, the final dSi concentrations in our biomimetic experiments were below the theoretical saturation limit of 2 mM. These observations suggest that R5 does not change the Si coordination unlike other organic compounds (e.g. disorbitol^40^, catechol^41^) and that Si concentration alone could be the dominant factor in Si isotopic fractionation within the diatom cell. It is important to note in this regard that R5 is only a simple mimic of a more complex biomolecule (silaffin-1A1). This natural silaffin contains extensive and unusual posttranslational modifications that are absent in R5^12^. Future work should explore whether natural silaffins can promote fractionation in a way that is not apparent with R5. The length of time required for the initial fractionation to revert to $\Delta ^{30}\text {Si}_\text {p-s}$ $\approx$ 0 is also concentration-dependent, being faster in samples with the highest initial concentrations of Si (Fig. 4). The initial concentration will impact the precipitation rates due to the concentration gradient and saturation of the supernatant. Nonetheless exchange is relatively slow, and presumably frustule biosilica is moved from the SDV to the cell exterior before such exchange behaviour can happen in vivo.

Intriguingly, the range of the kinetic $\Delta ^{30}\text {Si}_\text {p-s}$ values at day 0 and day 1 (− 0.58 $\textperthousand$ to − 3.34 $\textperthousand$, Fig. 4b) are in line with values of $\Delta ^{30}\text {Si}_\text {p-s}$ from field or culture studies of diatom silica^27,28,38,50^. These results imply that at least some of the variation found in these cellular studies comes from changes to Si concentration within the SDV. It is assumed that environmental conditions will influence the uptake and distribution of Si within the diatom cell^7^, and so this could lead to variability in Si concentration at the site of precipitation. It seems plausible that higher concentrations of dSi should give rise to greater fractionation within the frustule, and vice versa. A recent study^6^ suggested that dSi starvation can result in an increase in silicon content within the cell. This starvation response could potentially impact the isotopic composition of the resulting silica, by accentuating the kinetic fractionation effect. It would thus be of interest to examine the effect of dSi starvation on diatom isotopic fractionation, but to our knowledge such experiments have not yet been conducted.

In summary, our study confirms that Si isotopic fractionation in chemical experiments resembles that found in diatom silica from laboratory cultures and in the field. We also show that Si isotopic fractionation in the presence and absence of the R5 peptide is virtually indistinguishable. Our data confirm instead that the initial dSi concentration plays a major role in fractionation, with higher concentrations associated with increased $\Delta ^{30}\text {Si}_\text {p-s}$. The initial stage of precipitation drives Si isotopic fractionation, and in our model system this initial fractionation is gradually attenuated by chemical equilibrium processes. This leads to the proposition that the Si isotope fractionation factor in diatoms is not likely to be constant but instead is linked to a number of factors including external nutrients and internal cellular processes controlling dSi concentration within the cell. If the major control on internal dSi concentration is driven by the external environment, it is possible that diatom Si isotope archives used in palaeoenvironmental reconstructions could reveal more than just the biological consumption of dSi in surface oceans. They might also be useful means of understanding changes in diatom ecology due to environmental changes.

## Methods

### Experimental design

The media solution ND96 used during the experiment is composed of 47.8 g $\hbox {L}^{-1}$ NaCl, 1.48 g $\hbox {L}^{-1}$ KCl, 6.02 g $\hbox {L}^{-1}$ MgCl$_2$ and 5.95 g $\hbox {L}^{-1}$ HEPES. The solution was adjusted with HCl or NaOH to obtain a pH of 7.4 before the addition of sodium silicate. Sodium silicate solution (10% Na$_{2x}$O, 26.5% SiO$_2$, pH = 11.8) was added with different dilution factors to the ND96 buffer to cover a reasonable range of dSi concentrations to mimic the intra-cellular pools within diatoms^5,43^. The resulting concentrations being (743 mM, 371 mM, 148 mM, 74.3 mM, 37.1 mM, 18.6 mM, 7.4 mM and 3.7 mM). For all experiments and dilution factors, instantaneous precipitation was observed. pH was measured for the equilibrium (8 days) and kinetic (1 h) experiments only after the separation of the precipitate and the supernatant, and was not measured thereafter (Data available in Supplementary Table S1). For the biomimetic experiment, R5 was added to each sample at 0.01 g/ml (5 mM) before the addition of sodium silicate to ensure that the peptide is active^16^. The supernatant was sampled and separated from the precipitate at different times to evaluate the precipitation rates, solution/solid exchanges and the effect of the R5 peptide on the precipitation reactions and Si isotopic fractionation. The total volume for the abiotic and biomimetic was reduced to 1 ml due to the mass of R5 peptide available reducing the sub-sampling volume to 50 $\upmu$l. The analysis of other elements and pH was not possible for the abiotic and biomimetic experiments due to insufficient volume of supernatant solution. All experiments were carried out at room temperature, consistent with previous biomimetic experiments using R5^19,52^. Table 2 summarises the details of the four different experimental set-ups.

**Table 2:** Experimental design.

Experiment	[dSi] (mM)	Total volume (ml)	Interval	Total time
8 Days	743, 371, 148, 74.3, 37.1, 18.6, 7.4, 3.7	10	/	8 days
1 h	148, 74.3, 37.1, 18.6, 7.4, 3.7	10	/	1 h
Abiotic	148, 74.3, 37.1, 18.6	1	1 h, 1, 2, 4, 5, 6, 10 days	10 days
Biomimetic (R5)	148, 74.3, 37.1, 18.6	1	1 h, 1, 2, 4, 5, 6, 10 days	10 days

### Si concentration and isotopes analysis

dSi analyses of the supernatant were carried out after each time step. Centrifugation (3000*g* for 5 min) was carried before subsampling (at the surface of the supernatant) for dSi analysis to ensure no contamination from precipitate residue. dSi concentrations were measured using the silicomolybdate method^53^, using a Agilent Cary 60 UV-Vis spectrophotometer for 8 days, 1h and using a nanodrop ND 1000 for the abiotic experime nt, all at the wavelength of 410 nm. For the biomimetic (R5) experiment the dSi data have been extrapolated from the Multi Collector Induced Coupled Plasma Mass-Spectrometer (MC-ICP-MS) because R5 reacted with the colorimetric molybdate reagent. At the end of all experiment (total time), the supernatants and precipitates were separated by centrifugation. Precipitates were dissolved in 0.4N NaOH (Ananlar) at 100 $^{\circ }$C for 3 days and acidified with 6N HCl (in-house Teflon-distilled).

All samples were purified by cation exchange chromatography using Bio-Rad AG 50W $\times$ 12, 200–400 mesh in $\hbox {H}^{+}$ form resin. $\delta ^{30}\text {Si}$ analyses were carried out on the MC-ICP-MS (Finnigan Neptune s/n 1002, Bristol Isotopic Group). Measurement were operated on medium resolution and analysis were made on the low-mass side of the Si peaks where the polyatomic interferences (e.g. $^{14}\hbox {N}^{12}\hbox {O}$) were resolved from Si isotopes peaks. All sample analyses were at least duplicated and followed typical standard-sample bracketing and Mg doping methods^54^. The $\delta ^{30}\text {Si}_\text {dSi}$ results are reported relative to the standard NBS28 (Eq. 2). The measurement of the external standards LMG-08, with a mean value of -3.47 ${\pm }$ 0.17 $\textperthousand$ (2 s.d., n = 37) and Diatomite, with a mean value of 1.24 ${\pm }$ 0.19 $\textperthousand$ (2 s.d., n = 67) are in agreement with reference values^55,56^. For all samples and standards, the three isotopes ($^{28}$Si, $^{29}$Si, $^{30}$Si) were measured and results show good agreement with the mass-dependent fraction between $\delta ^{29}\text {Si}$ and $\delta ^{30}\text {Si}$ with $\delta ^{29}\text {Si}$ = 0.511 $\delta ^{30}\text {Si}$ (${\pm }$ 0.01).

2 $$\begin{aligned} \delta ^{x}\text {Si} (\textperthousand ) = \left( \frac{\left( \frac{^{x}\text {Si}}{^{28}\text {Si}}\right) _{sample}}{ \left( \frac{^{x}\text {Si}}{^{28}\text {Si}} \right) _{NBS28}} - 1 \right) \end{aligned}$$

with *x* corresponding to $^{29}$Si or $^{30}$Si and NBS28 being the international Si standard Quartz NBS28 (RM8546).

## Supplementary Information

Supplementary Information.

## Data Availability

All data are presented in the supplementary information document.
