# Supplementary material for: A biomimetic peptide has no effect on the isotopic fractionation during in vitro silica precipitation
Source: Sci Rep. 2021 May 6;11:9698. doi: 10.1038/s41598-021-88881-6 (PMC8102562; doi:10.1038/s41598-021-88881-6)
Supplement: Supplementary file 1 — Supplementary Information. [file 41598_2021_88881_MOESM1_ESM.pdf]

Supplementary information: A biomimetic peptide has no effect on the isotopic fractionation during *in vitro* silica precipitation

Lucie Cassarino[1,*], Paul Curnow[2,+], and Katharine R. Hendry[1,+]

[1]University of Bristol, School of Earth Sciences, Wills Memorial Building, Queen's Road, Brsitol, BS8 1RJ, UK
[2]University of Bristol, School of Biochemistry, Medical Sciences Building, University Walk, Bristol BS8 1TD, UK
[*]corresponding.l.cassarino@bristol.ac.uk
[+]these authors contributed equally to this work

ABSTRACT

The stable isotopic composition of diatom silica is used as a proxy for nutrient utilisation in natural waters. This approach provides essential insight into the current and historic links between biological production, carbon cycling and climate. However, estimates of isotopic fractionation during diatom silica production from both laboratory and field studies are variable, and the biochemical pathways responsible remain unknown. Here, we investigate silicon isotopic fractionation through a series of chemical precipitation experiments that are analogous to the first stages of intracellular silica formation within the diatom silicon deposition vesicle. The novelty of our experiment is the inclusion of the R5 peptide, which is closely related to a natural biomolecule known to play a role in diatom silicification. Our results suggest that the presence of R5 induces a systematic but non-significant difference in fractionation behaviour. It thus appears that silicon isotopic fractionation *in vitro* is largely driven by an early kinetic fractionation during rapid precipitation that correlates with the initial amount of dissolved silica in the system. Our findings raise the question of how environmental changes might impact silicon isotopic fractionation in diatoms, and whether frustule archives record information in addition to silica consumption in surface water.

Introduction

The data presented in this supplementary document are the data use for all the figures in the main text. Table 1 are the data corresponding to Figure 2 and Figure 3, Table 2 to the abiotic values of Figure 4 and Figure 5, and finally Table 3 to the biomimetic (R5) values of Figure 4 and Figure 5. Figure 1 show the evolution of dSi concentration over time from preliminary experiment. Table 4, 5, 6 are the supernatant and precipitate δ^{30}Sidata for the Equilibrium and Kinetic, Abiotic, and biomimetic experiments, respectively.

Table 1. Data corresponding to the Equilibrium and Kinetic experiments of Figure 2 and Figure 3 in the main text.

Figure	Experiment	Si addition	Time	dSi at t time (mM)	Si Loss	$\Delta^{30}Si_{p\text{-}s}$	2sd	pH
2 – 3	Equilibrium	743.0	8 days	444.0	0.40	0.02	0.24	10.9
2 – 3	Equilibrium	371.5	8 days	99.3	0.73	0.46	0.28	10.62
2 – 3	Equilibrium	148.6	8 days	2.7	0.98	-0.21	0.17	8.86
2 – 3	Equilibrium	74.3	8 days	2.4	0.97	-0.41	0.11	8.41
2 – 3	Equilibrium	37.2	8 days	2.4	0.94	-0.98	0.12	8.06
2 – 3	Equilibrium	18.6	8 days	2.3	0.87	-1.86	0.30	7.68
2 – 3	Equilibrium	7.4	8 days	2.4	0.68	-1.70	0.27	7.48
2 – 3	Equilibrium	3.7	8 days	2.5	0.34	-0.77	0.24	7.37
2 – 3	Kinetic	148.6	1 h	10.33	0.93	-2.71	0.09	7.85
2 – 3	Kinetic	74.3	1 h	6.69	0.91	-3.13	0.17	7.4
2 – 3	Kinetic	37.2	1 h	6.02	0.84	-1.55	0.22	7.72
2 – 3	Kinetic	18.6	1 h	5.45	0.71	-1.38	0.12	7.75
2 – 3	Kinetic	7.4	1 h	3.35	0.55	-0.46	0.09	7.55
2 – 3	Kinetic	3.7	1 h	1.98	0.47	-0.40	0.17	7.62

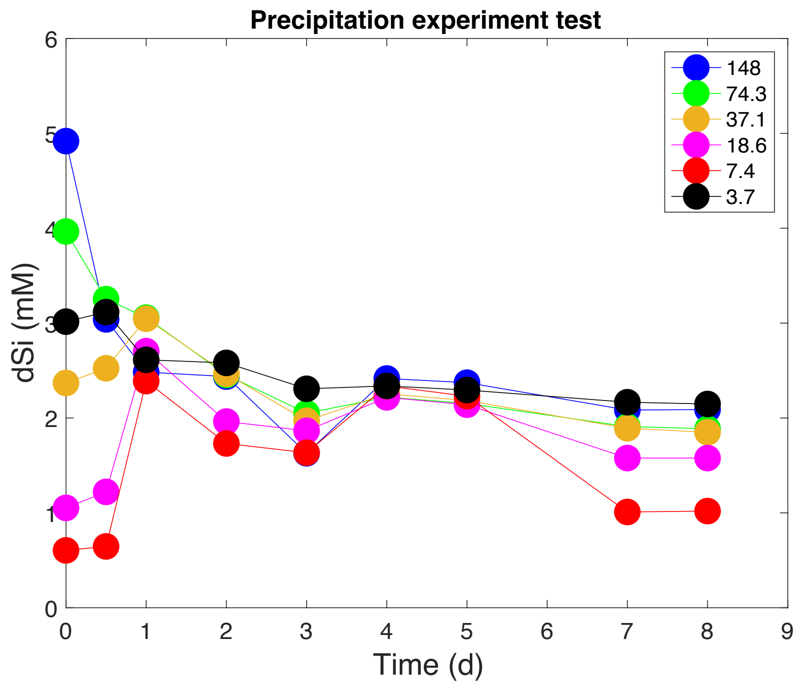

Figure 1. dSi precipitation rate from preliminary experiment to evaluate the equilibrium time.

Table 2. Data corresponding to the abiotic experiment of Figure 4 and Figure 5 in the main text.

Figure	Experiment	Si addition	Day	dSi at t time (mM)	Si Loss	$\Delta^{30}Si_{p-s}$	2sd
4 – 5	Abiotic		0	2.64	0.98	-3.34	0.22
4 – 5	Abiotic		1	2.82	0.98	-1.35	0.58
4 – 5	Abiotic		2	5.37	0.96	-0.72	0.21
4 – 5	Abiotic	148.6	4	3.19	0.98	-0.20	0.23
4 – 5	Abiotic		5	5.46	0.96	0.04	0.22
4 – 5	Abiotic		6	4.19	0.97	-0.01	0.26
4 – 5	Abiotic		10	3.83	0.97	0.16	0.18
4 – 5	Abiotic		0	2.64	0.96	-2.40	0.33
4 – 5	Abiotic		1	2.28	0.97	-1.20	0.10
4 – 5	Abiotic		2	4.55	0.94	-0.59	0.18
4 – 5	Abiotic	74.3	4	1.82	0.98	-0.26	0.13
4 – 5	Abiotic		5	5.28	0.93	0.02	0.19
4 – 5	Abiotic		6	2.64	0.96	-0.13	0.19
4 – 5	Abiotic		10	4.83	0.94	0.10	0.14
4 – 5	Abiotic		0	4.1	0.89	-2.01	0.26
4 – 5	Abiotic		1	2.19	0.94	-1.57	0.17
4 – 5	Abiotic		2	3.55	0.90	-0.90	0.71
4 – 5	Abiotic	37.2	4	4.65	0.87	-0.30	0.08
4 – 5	Abiotic		5	4.83	0.87	-0.15	0.11
4 – 5	Abiotic		6	3.83	0.90	-0.25	0.13
4 – 5	Abiotic		10	4.83	0.87	-0.08	0.14
4 – 5	Abiotic		0	2.28	0.88	-1.67	0.61
4 – 5	Abiotic		1	2.73	0.85	-1.45	0.34
4 – 5	Abiotic		2	3.83	0.79	-0.95	0.39
4 – 5	Abiotic	18.6	4	3.1	0.83	-0.66	0.51
4 – 5	Abiotic		5	5.28	0.72	-0.70	0.62
4 – 5	Abiotic		6	3.19	0.83	-0.41	0.40
4 – 5	Abiotic		10	2.82	0.85	-0.27	0.31

Table 3. Data corresponding to the biomimetic (R5) experiment of Figure 4 and Figure 5 in the main text.

Figure	Experiment	Si addition	Day	dSi at t time (mM)	Si Loss	$\Delta^{30}Si_{p\text{-}s}$	2sd
4 – 5	Biomimetic (R5)		0	0.92	0.99	-3.12	0.35
4 – 5	Biomimetic (R5)		1	/	/	-0.58	0.22
4 – 5	Biomimetic (R5)		2	1.7	0.99	-0.44	0.26
4 – 5	Biomimetic (R5)	148.6	4	1.43	0.99	-0.17	0.22
4 – 5	Biomimetic (R5)		5	1.44	0.99	0.04	0.32
4 – 5	Biomimetic (R5)		6	1.55	0.99	-0.17	0.23
4 – 5	Biomimetic (R5)		10	1.11	0.99	-0.02	0.27
4 – 5	Biomimetic (R5)		0	/	/	-2.45	0.15
4 – 5	Biomimetic (R5)		1	/	/	-1.03	0.35
4 – 5	Biomimetic (R5)		2	1.68	0.98	-0.73	0.14
4 – 5	Biomimetic (R5)	74.3	4	1.32	0.98	-0.38	0.21
4 – 5	Biomimetic (R5)		5	1.32	0.98	-0.28	0.17
4 – 5	Biomimetic (R5)		6	1.42	0.98	-0.35	0.14
4 – 5	Biomimetic (R5)		10	1.06	0.99	-0.19	0.21
4 – 5	Biomimetic (R5)		0	/	/	-1.83	0.12
4 – 5	Biomimetic (R5)		1	/	/	-1.61	0.04
4 – 5	Biomimetic (R5)		2	1.7	0.95	-1.00	0.02
4 – 5	Biomimetic (R5)	37.2	4	1.11	0.97	-0.77	0.12
4 – 5	Biomimetic (R5)		5	1.27	0.97	-0.89	0.02
4 – 5	Biomimetic (R5)		6	1.35	0.96	-0.57	0.02
4 – 5	Biomimetic (R5)		10	0.99	0.97	-0.38	0.10
4 – 5	Biomimetic (R5)		0	/	/	-1.45	0.26
4 – 5	Biomimetic (R5)		1	/	/	-1.32	0.20
4 – 5	Biomimetic (R5)		2	1.7	0.91	-1.23	0.17
4 – 5	Biomimetic (R5)	18.6	4	1.21	0.94	-0.97	0.35
4 – 5	Biomimetic (R5)		5	1.22	0.93	-0.68	0.17
4 – 5	Biomimetic (R5)		6	1.35	0.93	-0.64	0.19
4 – 5	Biomimetic (R5)		10	0.93	0.95	-0.34	0.20

Table 4. δ^{30}Sidata for all supernatant and precipitate of the equilibrium, kinetic experiments.

**Experiment**	**Si addition**	**Time**	**dSi at t time**	δ^{30}Sisupernatant	2sd	δ^{30}Siprecipitate	2sd
Equilibrium	743.0	8 days	444.0	0.08	0.17	0.11	0.17
Equilibrium	371.5	8 days	99.3	-0.26	0.14	0.20	0.24
Equilibrium	148.6	8 days	2.7	0.09	0.05	-0.12	0.16
Equilibrium	74.3	8 days	2.4	0.59	0.05	0.18	0.10
Equilibrium	37.2	8 days	2.4	0.96	0.09	-0.02	0.08
Equilibrium	18.6	8 days	2.3	1.59	0.26	-0.27	0.16
Equilibrium	7.4	8 days	2.4	1.28	0.23	-0.42	0.13
Equilibrium	3.7	8 days	2.5	0.52	0.20	-0.25	0.14
Kinetic	148.6	1 h	10.33	2.64	0.09	-0.07	0.02
Kinetic	74.3	1 h	6.69	2.46	0.11	-0.67	0.12
Kinetic	37.2	1 h	6.02	1.72	0.11	0.16	0.19
Kinetic	18.6	1 h	5.45	1.16	0.04	-0.22	0.11
Kinetic	7.4	1 h	3.35	0.37	0.03	-0.08	0.08
Kinetic	3.7	1 h	1.98	0.19	0.04	-0.21	0.17

Table 5. δ^{30}Sidata for all supernatant and precipitate of the abiotic experiments.

Experiment	Si addition	Time	dSi at t time	δ^{30}Sisupernatant	2sd	δ^{30}Siprecipitate	2sd
Abiotic		0	2.64	3.28	0.15	/	/
Abiotic		1	2.82	1.29	0.55	/	/
Abiotic		2	5.37	0.67	0.14	/	/
Abiotic	148.6	4	3.19	0.14	0.18	/	/
Abiotic		5	5.46	-0.09	0.16	/	/
Abiotic		6	4.19	-0.04	0.21	/	/
Abiotic		10	3.83	-0.21	0.09	-0.06	0.16
Abiotic		0	2.64	2.46	0.32	/	/
Abiotic		1	2.28	1.26	0.00	/	/
Abiotic		2	4.55	0.65	0.15	/	/
Abiotic	74.3	4	1.82	0.32	0.09	/	/
Abiotic		5	5.28	0.04	0.16	/	/
Abiotic		6	2.64	0.19	0.16	/	/
Abiotic		10	4.83	-0.04	0.10	0.06	0.10
Abiotic		0	4.1	1.99	0.24	/	/
Abiotic		1	2.19	1.55	0.15	/	/
Abiotic		2	3.55	0.88	0.70	/	/
Abiotic	37.2	4	4.65	0.28	0.01	/	/
Abiotic		5	4.83	0.14	0.07	/	/
Abiotic		6	3.83	0.23	0.10	/	/
Abiotic		10	4.83	0.07	0.12	-0.02	-0.08
Abiotic		0	2.28	1.59	0.55	/	/
Abiotic		1	2.73	1.37	0.21	/	/
Abiotic		2	3.83	0.86	0.28	/	/
Abiotic	18.6	4	3.1	0.57	0.43	/	/
Abiotic		5	5.28	0.61	0.56	/	/
Abiotic		6	3.19	0.32	0.29	/	/
Abiotic		10	2.82	0.18	0.16	-0.09	-0.27

Table 6. δ^{30}Sidata for all supernatant and precipitate biomimetic experiments.

Experiment	Si addition	Time	dSi at t time	δ^{30}Sisupernatant	2sd	δ^{30}Siprecipitate	2sd
Biomimetic (R5)		0	0.92	3.05	0.27	/	/
Biomimetic (R5)		1	/	0.51	0.03	/	/
Biomimetic (R5)		2	1.7	0.38	0.15	/	/
Biomimetic (R5)	148.6	4	1.43	0.10	0.04	/	/
Biomimetic (R5)		5	1.44	-0.10	0.23	/	/
Biomimetic (R5)		6	1.55	0.11	0.06	/	/
Biomimetic (R5)		10	1.11	-0.04	0.16	-0.06	0.22
Biomimetic (R5)		0	/	2.45	0.04	/	/
Biomimetic (R5)		1	/	1.04	0.31	/	/
Biomimetic (R5)		2	1.68	0.73	0.00	/	/
Biomimetic (R5)	74.3	4	1.32	0.38	0.15	/	/
Biomimetic (R5)		5	1.32	0.29	0.08	/	/
Biomimetic (R5)		6	1.42	0.36	0.02	/	/
Biomimetic (R5)		10	1.06	0.19	0.16	0.00	0.14
Biomimetic (R5)		0	/	1.71	0.12	/	/
Biomimetic (R5)		1	/	1.49	0.03	/	/
Biomimetic (R5)		2	1.7	0.88	0.00	/	/
Biomimetic (R5)	37.2	4	1.11	0.64	0.12	/	/
Biomimetic (R5)		5	1.27	0.77	0.00	/	/
Biomimetic (R5)		6	1.35	0.44	0.01	/	/
Biomimetic (R5)		10	0.99	0.26	0.10	-0.12	0.02
Biomimetic (R5)		0	/	1.45	0.20	/	/
Biomimetic (R5)		1	/	1.32	0.11	/	/
Biomimetic (R5)		2	1.7	1.23	0.04	/	/
Biomimetic (R5)	18.6	4	1.21	0.97	0.31	/	/
Biomimetic (R5)		5	1.22	0.68	0.00	/	/
Biomimetic (R5)		6	1.35	0.64	0.10	/	/
Biomimetic (R5)		10	0.93	0.34	0.12	0.00	0.17
